# Case Report: ISG15 deficiency caused by novel variants in two families and effective treatment with Janus kinase inhibition

**DOI:** 10.3389/fimmu.2023.1287258

**Published:** 2023-12-05

**Authors:** Alice Burleigh, Elena Moraitis, Eman Al Masroori, Eslam Al-Abadi, Ying Hong, Ebun Omoyinmi, Hannah Titheradge, Karen Stals, Wendy D. Jones, Anthony Gait, Vignesh Jayarajan, Wei-Li Di, Neil Sebire, Lea Solman, Malobi Ogboli, Steven B. Welch, Annapurna Sudarsanam, Ian Wacogne, Fiona Price-Kuehne, Barbara Jensen, Paul A. Brogan, Despina Eleftheriou

**Affiliations:** ^1^ Infection, Immunity and Inflammation Department, University College London Great Ormond Street Institute of Child Health, London, United Kingdom; ^2^ Centre for Adolescent Rheumatology Versus Arthritis at University College London (UCL), London, United Kingdom; ^3^ Paediatric Rheumatology Department, Great Ormond Street Hospital for Children NHS Foundation Trust, London, United Kingdom; ^4^ Department of Rheumatology, Birmingham Women’s and Children’s NHS Foundation Trust, Birmingham, United Kingdom; ^5^ Clinical Genetics, Birmingham Women’s and Children’s NHS Foundation Trust, Birmingham, United Kingdom; ^6^ Clinical Sciences Department, University of Birmingham, Birmingham, United Kingdom; ^7^ Exeter Genomics Laboratory, Royal Devon and Exeter NHS Foundation Trust, Exeter, United Kingdom; ^8^ Clinical Genetics, Great Ormond Street Hospital for Children NHS Foundation Trust, London, United Kingdom; ^9^ Molecular and Cellular Immunology Unit, University College London (UCL), Great Ormond Street Institute of Child Health, London, United Kingdom; ^10^ Histopathology Department, Camelia Botnar Laboratories, Great Ormond Street Hospital, London, United Kingdom; ^11^ Department of Dermatology, Great Ormond Street Hospital for Children NHS Foundation Trust, London, United Kingdom; ^12^ Department of Dermatology, Birmingham Women’s and Children’s NHS Foundation Trust, Birmingham, United Kingdom; ^13^ Department of Paediatrics, Heartlands Hospital, University Hospitals Birmingham, Birmingham, United Kingdom; ^14^ Department of Paediatric Neurology, Birmingham Women’s and Children’s NHS Foundation Trust, Birmingham, United Kingdom; ^15^ Department of General Paediatrics, Birmingham Women's and Children's NHS Foundation Trust, Birmingham, United Kingdom

**Keywords:** ISG15 deficiency, ISG15, interferonopathy, microdeletion, Janus kinase inhibition, baricitinib, whole exome sequencing, interferon

## Abstract

ISG15 deficiency is a rare disease caused by autosomal recessive variants in the *ISG15* gene, which encodes the ISG15 protein. The ISG15 protein plays a dual role in both the type I and II interferon (IFN) immune pathways. Extracellularly, the ISG15 protein is essential for IFN-γ-dependent anti-mycobacterial immunity, while intracellularly, ISG15 is necessary for USP18-mediated downregulation of IFN-α/β signalling. Due to this dual role, ISG15 deficiency can present with various clinical phenotypes, ranging from susceptibility to mycobacterial infection to autoinflammation characterised by necrotising skin lesions, intracerebral calcification, and pulmonary involvement. In this report, we describe novel variants found in two different families that result in complete ISG15 deficiency and severe skin ulceration. Whole exome sequencing identified a heterozygous missense p.Q16X *ISG15* variant and a heterozygous multigene 1p36.33 deletion in the proband from the first family. In the second family, a homozygous total *ISG15* gene deletion was detected in two siblings. We also conducted further analysis, including characterisation of cytokine dysregulation, interferon-stimulated gene expression, and p-STAT1 activation in lymphocytes and lesional tissue. Finally, we demonstrate the complete and rapid resolution of clinical symptoms associated with ISG15 deficiency in one sibling from the second family following treatment with the Janus kinase (JAK) inhibitor baricitinib.

## Introduction

The range of autoinflammatory and immunodysregulatory disorders caused by inborn errors in the type I and II interferon (IFN) pathways has significantly expanded in recent years (1). Type I interferonopathies are the result of harmful variants in various genes that encode proteins involved in DNA damage sensing, the proteasome, the endoplasmic reticulum-golgi apparatus axis, or proteins directly involved in IFN-I receptor signaling. Skin vasculitis and neurological involvement, including cerebral calcification, are common features (2). On the other hand, genetic disorders affecting the type II interferon pathway are associated with Mendelian susceptibility to mycobacterial disease (MSMD) (3), which can also manifest as a reaction to the Bacillus Calmette-Guérin (BCG) vaccine. Recently, interferon-stimulated gene 15 (ISG15) deficiency has been identified as a complex disorder with a mixed phenotype that encompasses features of both type I and type II interferonopathies (4–6). This is not surprising considering the dual role of ISG15 in these pathways.

ISG15 deficiency remains an extremely rare disease, with fewer than 100 cases reported worldwide. In this report, we expand the genotypic spectrum of ISG15 deficiency by describing two families, one of White British and one of Pakistani ancestry, with novel microdeletion and nonsense variants in *ISG15* that result in complete ISG15 deficiency. Additionally, we demonstrate for the first time the efficacy of Janus kinase (JAK) inhibitor baricitinib as a treatment for this disease.

## Materials and methods

### Study participants

We obtained written informed consent from all participants and controls (ethics no. 08H071382 and 11/LO/0330) who participated in the study.

### Whole exome sequencing and analysis

DNA was extracted from EDTA blood using the Gentra Puregene Blood Kit (Qiagen). For family A, DNA was sent to Nonacus/Informed Genomics Ltd for WES using their ExomeCG Cell3™ enrichment technology and data processing services. Reads were aligned to GRCh38 using BWA-MEM (7), and genotyping was performed with Sentieon^®^ DNAseq^®^. Data were then annotated using wANNOVAR (8) and filtered in-house. Variants were classified according to the ACMG/AMP and ACGS guidelines (9, 10). Exomiser 12.1.0 (11) was also applied to prioritise variants associated with the described clinical phenotype. Copy number variant (CNV) analysis was performed using ExomeDepth 1.1.10 (12). For family B, trio WES and subsequent variant calling/filtering was carried out as previously described (13). CNV calling was undertaken using SavvyCNV (14).

### Sanger sequencing

The *ISG15* nonsense variant in family A was confirmed by PCR and Sanger sequencing using the following primers (Merck) to amplify and sequence *ISG15* exon 2 forward: 5’-GTAGAGGACAGACAGGAGGG-3’ and reverse: 5’-ATCTTCTGGGTGATCTGCGC-3’.

### Targeted genomic microarray analysis

Targeted genomic microarray analysis was performed through Great Ormond Street Hospital clinical services using the Illumina Beadchip CytoSNP850K platform and infoQuant Fusion v7 software. At Birmingham Women’s and Children’s Hospital, the Illumina GSAv3 microarray was applied, then analysis performed in build GRCh37 using NxClinical v6.0 (BioDiscovery) and FASST2 CNV calling algorithm.

### Peripheral blood mononuclear cells isolation and western blotting for ISG15 protein

Peripheral blood mononuclear cells (PBMC) were isolated from freshly drawn heparinised blood using gradient density centrifugation with Lymphoprep™. Cells were stimulated with 1000U/mL IFNα2b (GenScript) for 24 hours. Cells were lysed with RIPA buffer (Thermo Fisher Scientific) with 1% protease inhibitor cocktail (Roche), then protein was quantified by BCA assay and normalised to 10µg. Lysates were boiled at 95°C for 5 mins with Laemmli buffer (Bio-Rad). After SDS-PAGE and transfer, membranes were blocked with 5% milk, then probed with primary antibodies against ISG15 (F-9; Santa Cruz Biotechnology) or β-actin (MAB1501R; Merck Millipore), followed by goat anti-mouse IgG, HRP secondary antibody (Thermo Fisher Scientific). Signal was detected using Amersham ECL Western Blotting Detection Reagent (Cytiva). Images were taken using a ChemiDoc Imager (Bio-Rad) and analysed using Image Lab software (Bio-Rad).

### Quantitative PCR of interferon stimulated genes

Blood was collected into PAXgene^®^ tubes (PreAnalytix), and RNA extracted using PAXgene^®^ Blood RNA kit 50 v2 (PreAnalytix). Single-strand cDNA was generated using High-Capacity cDNA reverse transcription kit (Applied Biosystems). qPCR was then performed using iTaq Universal SYBR Green Supermix (Bio-Rad) and the relevant QuantiTect Primer Assays (QIAGEN). The relative abundance of 11 targets (*CXCL10, CXCL9, IFI27, IFI44L, IFIT1, IFNB1, IFNG, IFNL1, IL18, RSAD2, SIGLEC1*) was normalised to the expression level of *ACTB*, assessed using Bio-Rad CFX Manager software.

### Cytokine and interferon assays

Cytokine levels were measured in plasma or serum using V-PLEX proinflammatory panel 1 (TNF-α, IL-6, IL-8, IL-1β, IFN-γ); V-PLEX chemokine panel 1 (MCP-1, IP-10); S-PLEX human IFN-α2a; and S-PLEX human IFN-β Meso Scale Discovery kits (Meso Scale Diagnostics) according to the manufacturer’s instructions.

### STAT1 phosphorylation assay

PBMC were stimulated with 2000U/mL IFNα2b (GenScript) for 0, 10, 20 and 30 mins. Cells were fixed for 10 mins at 37°C with Phosflow™ Fix Buffer I (BD), then permeabilised for 30 mins at RT with Perm Buffer III (BD). Cells were then incubated with PE-anti-STAT1 pY701 (BD) for 45 mins and then analysed using the CytoFLEX (Beckman Coulter). Data were analysed using FlowJo (BD).

### Immunofluorescence staining for p-STAT1 in lesional skin tissue

Paraffin embedded skin histopathological sections were dewaxed by submerging slides in six histological staining boxes for 5 mins each, in the order xylene x2, 100% ethanol x2, 70% ethanol x2. Slides were then boiled in citrate buffer for 15 mins, blocked in 3% BSA in PBS for 30 mins, then incubated at 4°C overnight with primary antibody (anti-p-STAT1 pY701, Cell Signaling, 9167). Slides were then incubated with secondary antibody (anti-rabbit IgG-Alexa Fluor 555, Invitrogen, A-21429) for 1 hour at RT. Slides were incubated with DAPI (5µg/ml in PBS) for 2 mins, then mounted using mounting medium for fluorescence (Vectashield) and imaged using a ZEISS LSM710 inverted confocal microscope (Carl Zeiss Ltd, UK). Images were analysed in ImageJ (NIH).

## Results

### Clinical presentation

**Figure 1:**
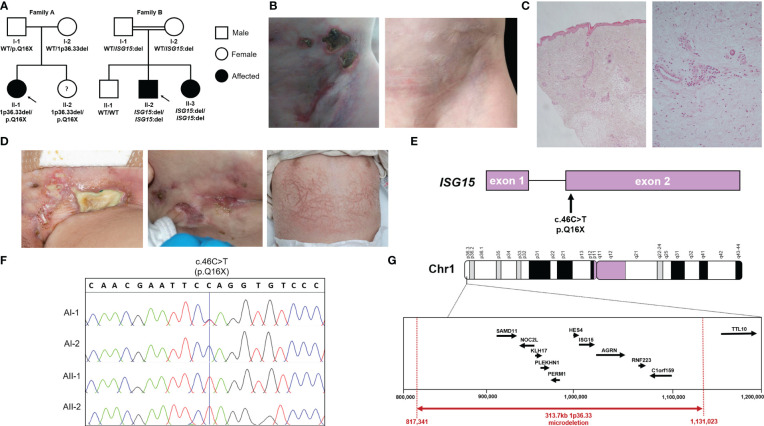
Pedigrees, *ISG15* variants and clinical phenotype of ISG15 deficiency. **(A)** Pedigrees and familial segregation of the *ISG15* alleles with affected individuals marked in black and unaffected in white. Note AII-2 is currently asymptomatic. **(B)** AII-1 experienced extensive ulcerative skin lesions observed in the groin and lower abdominal wall, which left persistent scarring and lipoatrophy after resolution of the lesions. **(C)** Photomicrographs of skin biopsy from AII-1 at 1-year-of-age demonstrating a moderate diffuse dermal perivascular inflammatory infiltrate composed of predominantly mononuclear inflammatory cells. There is focal extension into subcuticular adipose tissue but no active panniculitis in this biopsy (H&E original magnifications x20 LT and x100 RT). **(D)** BII-2 experienced severe ulcerative skin lesions observed in the groin and neck, as well as livedoid rash with prominent skin capillaries. **(E)** Schematic localisation of the c.46C>T variant in *ISG15* gene found in family A. **(F)** Sanger sequencing traces showing c.46C>T variant in members of family A. **(G)** Map of chromosome 1p36.33 region showing microdeletion variant encompassing *ISG15* found in family A.

AII-2 is a 4-year-old female who was born to healthy White British non-consanguineous parents at term (**Figure 1A**). At the age of 5 months, she presented with ulcerative skin lesions in her right groin and left axilla, along with extensive necrosis and, later, lipoatrophy (**Figure 1B**). No other symptoms were reported, and there were no signs of neurological abnormalities. Extensive investigations yielded unremarkable results (**Table 1**). Candida species was isolated from a single skin swab, which was treated with topical antifungal creams but showed no significant improvement. A brain computed tomography (CT) scan did not reveal any intracerebral calcifications. Skin histology demonstrated fibrosis of the subcutaneous layer, mild lobular panniculitis, and superficial perivascular inflammatory infiltrate (**Figure 1C**). Based on these findings, an autoinflammatory disease causing extensive skin ulceration was suspected, and genetic testing was requested. Treatment included topical steroids and two short courses of oral prednisolone (1mg/kg for 5-7 days), resulting in modest and temporary improvement of the skin inflammation. Methotrexate (15mg/m^2^ subcutaneously) was also initiated at 21 months of age. This led to significant improvement, with complete resolution of skin ulceration but persistent scarring and lipoatrophy. After 18 months, methotrexate treatment was discontinued. Currently, the patient remains well, without any recurrent skin lesions or notable symptoms, and her development continues to be appropriate for her age.

**Table 1:** Clinical and laboratory features associated with ISG15 deficiency.

	AII-1	BII-2	BII-3
Demographics			
**Sex**	Female	Male	Female
**Age at first presentation**	5 months	2 months	6 months
**Ethnicity**	White British	Pakistani	Pakistani
Clinical features			
**Skin manifestations** **Area of body affected** **Necrosis** **Ulceration** **Livedoid features** **Hyperpigmentation** **Scarring** **Photosensitivity**	Groin/abdomen Yes Yes Yes Yes Yes No	Groin/abdomen/torso/neck/axillae Yes Yes Yes Yes Yes No	Axillae No Yes No No Yes No
**Arthritis**	No	No	No
**Myalgia**	No	No	No
**Recurrent fevers**	No	Yes	Yes
**Raynaud’s phenomenon**	No	No	No
**Neurological involvement**	None	Mild encephalopathy Hypotonia Seizures	No
**Lung involvement**	None	Recurrent respiratory tract infections	Asymptomatic
**Development**	Normal	Global developmental delay	Mild gross motor delay
**Growth**	Normal	<3^rd^ Centile for Height and weight (25-50^th^ at birth)	<3^rd^ Centile for Height and weight (9-25^th^ at birth)
**History of recurrent infections** **History of mycobacterial infection** **BCG vaccination reaction**	Recurrent UTIs No documented mycobacterial infections Not had BCG vaccination	Recurrent lower respiratory tract infections. Groin lesions cultured faecal organisms and Candida as below, but were likely contaminants Skin lesions were treated empirically for atypical Mycobacteria, but no microbiological confirmation Normal BCG scar	No Negative Normal BCG scar
Investigations			
Imaging			
**Neuroimaging**	CT brain - normal	CT brain - normal MRI brain – plagiocephaly	Not done
**Abdominal ultrasound**	Normal	Hepatosplenomegaly	Not done
**Chest radiograph**	Normal	Persistent bilateral perihilar bronchial wall thickening with patchy inflammatory changes, episodic unilateral/bilateral consolidation and/or effusions	Bilateral peribronchial thickening with no effusions or consolidation (incidental finding during pre-treatment chest radiograph screening)
Microbiology			
**Skin swab culture**	Candida Lusitania	Candida parapsilosis Enterobacter cloacae Klebsiella pneumoniae ESBL *E. coli*	Not done
**Blood and CSF cultures**	Not done	Negative	Not done
**Respiratory specimens**	Not done	SARS-Cov-2 and parainfluenza (NPA) Respiratory syncytial virus and klebsiella pneumoniae (BAL)	Not done
Blood tests (RR*)			
**Haemoglobin *(105-135 g/L)***	109-125 g/L	88-130 g/L	109-119g/L
White blood cell (5-15 x10*9/L)	6.6-11.45 x10*9/L	3.3-19.9 x10*9/L	6.6-7.9 x 10*9/L
Neutrophils (1.5-8.5 x10*9/L)	1.9-2.3 x10*9/L	1.4-13.3 x10*9/L	1.5-2.6 x 10*9/L
Lymphocytes (2-9.5 x10*9/L)	3.7-8.15 x10*9/L	1.0-5.7 x10*9/L	3.7-4.8 x 10*9/L
Platelets (150-450 x10*9/L)	301-341 x10*9/L	121-468 x10*9/L	307-421 x 10*9/L
**CRP *(0-20mg/L)***	<5-17 mg/L	<1-14 mg/L	<1mg/L
**ESR *(0-10 mm/hr)***	5-12 mm/hr	2-19 mm/hr	8-12mm/hr
**Serum amyloid A *(<10mg/L)***	<3.5mg/L	Not done	<3.2mg/L
**Ferritin *(8.6-74.0 ug/L)***	Not done	305-3135 ug/L	Not done
**LDH *(192-321 U/L)***	Not done	857-1107 U/L	Not done
**Triglycerides *(0.36-1.31 mmol/L)***	Not done	2.1-6.27 mmol/L	0.56mmol/L
**Liver function tests** **ALT *(5-45 U/L)*** **AST *(20-60 U/L)*** **GGT *(6-19 U/L)***	20-30 U/L 56 U/L 10 U/L	48-147 U/L 61-169 U/L 33-45 U/L	20-24 U/L Not done Not done
**Autoantibodies and immunology tests** **ANA/dsDNA/ENA** **Immunoglobulin levels** **Pneumococcal and tetanus vaccine response** **Anticardiolipin antibodies (0-17 GPL U/ml)** **Lymphocyte subsets** **PHA responses** **Complement function** **C3 *(0.75-1.65 g/L)*** **C4 *(0.14-0.54 g/L)***	Negative Normal Normal 15.3 GPLU/ml Normal distribution Normal Normal 1.26 g/L 0.28 g/L	Negative Normal Normal 4.0 GPL U/ml Normal distribution Not done Normal (activated) 1.77 g/L >0.58 g/L	Negative Normal Normal Not done Not done Not done Not done

*RR Reference ranges as per GOSH laboratory. Values provided indicate lower and higher result obtained for specific tests. Abbreviations: UTI, urinary tract infections; CT, computed tomography; MRI, magnetic resonance imaging; CSF, cerebrospinal fluid; ESBL E.coli, extended spectrum beta-lactamase-producing Escherichia coli; RR, reference range; CRP, C-reactive protein; ESR, erythrocyte sedimentation rate; LDH, lactate dehydrogenase; ALT, alanine transaminase; AST, aspartate transaminase; GGT, gamma glutamyl transferase; ANA, anti-nuclear antibody; dsDNA, anti-double-stranded DNA antibodies; ENA, extractable nuclear antigen; C3, component 3; C4, component 4; TB, tuberculosis; BCG, Bacillus Calmette-Guerin; PHA, phytohaemagglutinin; GPL, G phospholipid; NPA, Nasopharyngeal aspirates; BAL, Bronchoalveolar lavage.

BII-2 is a 2-year-old male born to consanguineous parents of Pakistani ancestry (**Figure 1A**). He first presented at 2 months of age with ulcerated skin lesions on his neck (**Figure 1D**). Topical antibiotics showed minimal response, but he remained systemically well until 8 months of age when he experienced status epilepticus accompanied by fever and vomiting. At that time, a livedoid rash was observed on his torso, along with further ulcerated skin lesions in his groin. Skin biopsy revealed widened subcutaneous septae containing neutrophils and fibrin, and fat necrosis with a hyalinised appearance, raising suspicion of an atypical mycobacterial infection. The T-SPOT.TB tuberculosis test yielded an indeterminate result, and cerebrospinal fluid (CSF) analysis showed elevated protein levels of 0.99g/L (reference range: 0.15-0.45g/L), prompting initiation of empirical anti-TB treatment along with antiepileptic medication. However, CT and magnetic resonance imaging (MRI) showed no evidence of meningitis and CSF grew no Mycobacteria. A 12-month course of ethambutol and ciprofloxacin was completed to cover possible atypical Mycobacterial skin infection, but there was no microbiological confirmation of this. Extensive autoimmune and immunology tests mostly returned negative results (**Table 1**). Over the following 18 months, the patient experienced recurrent seizures, fevers, and worsening skin lesions. He also had recurrent respiratory tract infections with chest radiograph changes, and global developmental delay was noted. CT brain scans showed no intracranial calcifications, and MRI scans of the brain revealed no intracerebral inflammation but did note plagiocephaly. During one of these episodes requiring hospital admission, the patient developed pancytopenia, hypertriglyceridemia, and hyperferritinemia, prompting a bone marrow aspirate examination that showed reactive changes without evidence of malignancy or hemophagocytic lymphohistiocytosis (HLH). A repeat T-SPOT.TB test was negative but considering the clinical picture possibly suggestive of mycobacterial skin infection, genetic analysis for Mendelian susceptibility to TB was conducted, revealing a homozygous *ISG15* gene deletion (see details of genetic testing below). The patient was subsequently initiated on oral prednisolone 1mg/kg/day tapered over 8-10 weeks and the JAK inhibitor baricitinib (2mg orally twice daily). This treatment led to marked clinical improvement, with no further febrile illnesses or seizures and complete resolution of ulcerated skin lesions. The patient’s developmental skills have improved, with only mild speech and cognitive delay. BII-3, the younger sister of BII-2, recently developed a single skin ulcerative lesion and mild developmental delay and she will soon be started on baricitinib (2mg twice daily).

A summary of all the presenting clinical features and laboratory/imaging investigations for index cases in family A (AII-1) and B (BII-2 and BII-3) is shown in **Table 1**.

### Genetic analysis

Both families underwent whole exome sequencing (WES).

In family A, we identified a novel nonsense variant in *ISG15* on chromosome 1 exon 2 c.46C>T (p.Q16X) (**Figure 1E**). Interestingly, this variant was observed in a homozygous state in both AII-1 and her younger sister AII-2, however only their father AI-1 was heterozygous for this variant. Sanger sequencing of *ISG15* exon 2 in the family confirmed this finding (**Figure 1F**). Since this did not explain the inheritance of the homozygous variant in both siblings, we considered the possibility of a heterozygous deletion at the same locus in the mother and both siblings. Using ExomeDepth (12) to assess copy number variants (CNV) detected from WES data, we identified a heterozygous deletion encompassing *ISG15* and a surrounding nine genes (*SAMD11, NOC2L, KLHL17, PLEKHN1, PERM1, HES4, ISG15, AGRN, RNF223, C1orf159*) in the mother (AI-2) and both siblings (AII-1 and AII-2), but not the father (AI-1). Since this analysis method only included exonic regions, we next applied targeted genomic microarray analysis to accurately assess the deletion size, which confirmed the presence of a heterozygous 313.7kb deletion at the expected locus (chr1:817,341-1,131,023) (**Figure 1G**).

In Family B, trio WES analysis with SavvyCNV identified a homozygous complete *ISG15* deletion in the proband BII-2 with biparental inheritance. This was a single gene deletion as WES coverage of the surrounding genes was as expected. Upon investigation of the siblings using ExomeDepth, we found that younger sister BII-3 also had homozygous deletion of *ISG15*, however brother BII-1 had two wildtype variants. Of note, earlier microarray analysis in the proband did not detect this deletion, due its small size (∼1kb).

### Absence of ISG15 protein production in PBMC from individuals with biallelic mutant *ISG15* alleles

**Figure 2:**
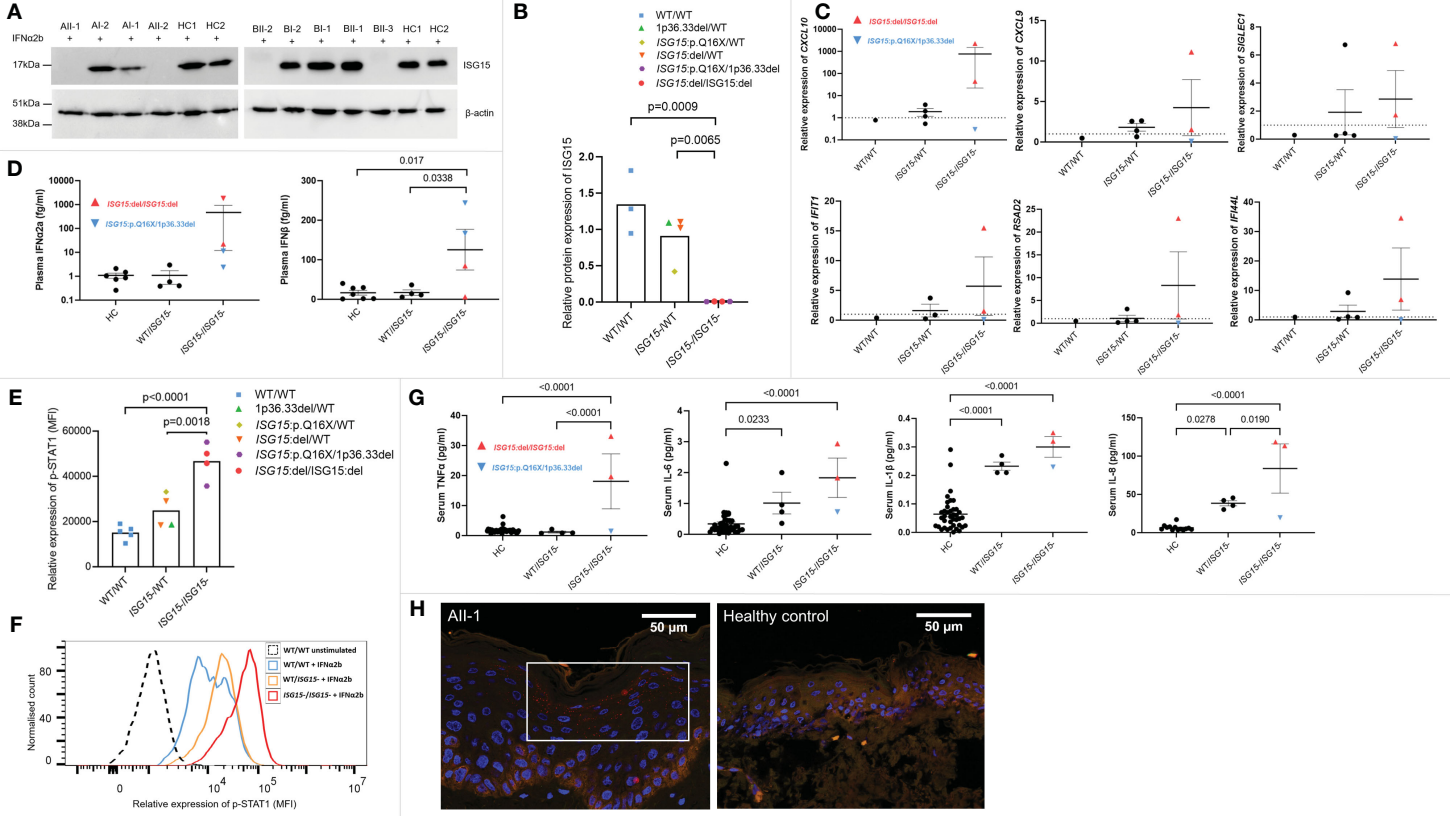
ISG15 protein expression and IFN gene expression, p-STAT1 signalling and cytokines in ISG15 deficiency. **(A, B)** Representative western blots and densitometry analysis of ISG15 protein levels detected in peripheral blood mononuclear cells (PBMC) isolated from all individuals in families A and B, and two healthy controls (HC1 and HC2). PBMC were stimulated with 1000U/mL IFN-α2b for 24h, and then lysed. β-actin was used as a loading control. There was no ISG15 protein detected in PBMC from individuals AII-1 and AII-2 carrying the *ISG15* p.Q16X/1p36.33del variants; similarly no protein was detected in PBMC from BII-2 and BII-3 with *ISG15*:del/*ISG15*:del. **(C)** Gene expression of interferon stimulated genes (ISGs) *CXCL10*, *CXCL9*, *SIGLEC1*, *IFIH1*, *RSAD2* and *IFI44L* were measured using qPCR in all members of family A and B, excluding AII-2. **(D)** Plasma concentrations of IFN-α2a and IFN-β were measured using Meso Scale Discovery (MSD) assays in all members of both families. Both IFN-α2a and IFN-β were raised in individuals with ISG15 deficiency, compared with both heterozygous carriers and healthy controls. **(E)** Relative expression of p-STAT1 (MFI, median fluorescence intensity) in lymphocytes derived from members of families A and B and healthy controls after 10 mins IFN-α2b stimulation. Ten mins was selected as a representative time point, although similar trends were observed at all time points. **(F)** Representative flow cytometric histogram showing p-STAT1 expression (MFI) in lymphocytes of individuals with no, heterozygous, and homozygous loss of ISG15 after 10 mins IFN-α2b stimulation. **(G)** Serum concentrations of proinflammatory cytokines TNF-α, IL-6, IL-1β and IL-8 were measured using Meso Scale Discovery (MSD) assays. All were significantly raised in individuals with ISG15 deficiency, compared with healthy controls. **(H)** Immunofluorescence staining for p-STAT1 (red) was performed on a skin biopsy from AII-1 and a healthy control. In AII-1, p-STAT1 is visible as small red spots in the cytoplasm of differentiating keratinocytes, which is not visible in the healthy control epidermis. For all graphs, averages are shown as mean +-SEM. P values were calculated by one-way ANOVA with multiple comparisons. Data are grouped by functional *ISG15* zygosity.

To investigate how these three novel variants in *ISG15* affect ISG15 protein expression, PBMC from all members of both families and healthy controls were stimulated with IFNα2b to induce ISG15 expression, then ISG15 protein levels were analysed with western blotting. Individuals AII-1, AII-2, BII-2, and BII-3 showed complete deficiency of ISG15 protein (**Figures 2A, B**), consistent with these individuals possessing biallelic total deletion or early truncation variants in *ISG15*. As expected, heterozygous carriers of *ISG15* variants (AI-1, AI-2, BI-1, and BI-2) showed decreased levels of ISG15 when compared with the healthy controls (**Figure 2B**).

### Type I interferon stimulated gene expression, p-STAT1 signalling and circulating cytokines in ISG15 deficient individuals

Patients with type I interferonopathies frequently display increased expression of interferon-stimulated genes (ISGs), and elevated levels of type I interferons in the blood. We noted high expression of ISGs in ISG15 deficient individuals, particularly in BII-2 who was the only individual with active disease at the time of blood sampling (**Figure 2C**). Interestingly, the ISG15 deficient individuals did not show altered expression of type II interferon *IFNG* when compared with parent carriers (p=0.75) or wildtype BII-1 (p=1.0). Similarly, type III interferon *IFNL1* expression was also not raised in patients compared with carriers (p=0.12) or BII-1 (p=0.97). The patients also showed elevated plasma IFN-α2a and IFN-β compared with both carriers and healthy controls (**Figure 2D**). Since ISG15 plays a crucial role in negatively regulating type I interferon signalling, we next explored this pathway by examining p-STAT1 expression in lymphocytes of all family members. We found that all four ISG15 deficient individuals showed markedly higher levels of p-STAT1 than both the healthy control (p<0.0001) and carrier (p=0.0018) groups (**Figures 2E, F**). We also tested serum levels of proinflammatory cytokines, and observed significantly raised levels of TNF-α, IL-1β, IL-6 and IL-8 in the ISG15 deficient individuals compared with healthy controls (**Figure 2G**).

### p-STAT1 activation in lesional skin tissue obtained from AII-1

In line with previous reports (6), we next explored whether there was STAT1 activation in lesional skin tissue obtained from AII-1. We confirmed upregulation of p-STAT1 expression in the differentiated keratinocytes in the epidermis of AII-1 that was not observed in the healthy control (**Figure 2H**).

### Janus kinase inhibition with baricitinib for ISG15 deficiency

**Figure 3:**
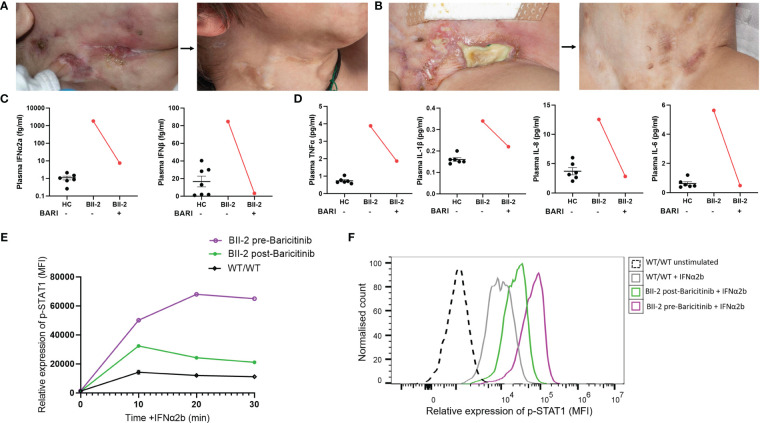
Janus kinase inhibition with baricitinib in the treatment of ISG15 deficiency. **(A)** Improvement of neck and **(B)** groin ulcerative lesions in BII-2 after six-week course of baricitinib. **(C)** Plasma concentrations of IFN-α2a and IFN-β before and after treatment with baricitinib, measured by Meso Scale Discovery (MSD) assay, compared with healthy controls (HC). **(D)** Plasma concentrations of TNF-α, IL-6, IL-1β and IL-8 before and after treatment with baricitinib, measured by Meso Scale Discovery (MSD) assay, compared with healthy controls (HC). **(E)** There was downregulation of p-STAT1 (median fluorescence intensity, MFI) expression in IFN-α2b treated lymphocytes derived from BII-2 post-treatment with baricitinib compared to baseline (pre-treatment) levels of expression. **(F)** Representative flow cytometric histogram showing p-STAT1 expression (MFI) in lymphocytes derived from BII-2 stimulated for 30 mins with IFN-α2b examined before treatment with baricitinib was started and post-treatment. For all graphs, averages are shown as mean +-SEM.

BII-2 was started on treatment with the JAK inhibitor baricitinib in combination with oral prednisolone (1mg/kg/day weaned over 8-10 weeks). This resulted in resolution of all cutaneous lesions (**Figures 3A, B**) and other clinical symptoms, and prednisolone therapy has been weaned off. Peripheral proinflammatory cytokines and type I interferon levels reduced after treatment (**Figures 3C, D**). Expression of p-STAT1 levels in IFN-α2b-treated lymphocytes was also significantly reduced at six-week post initiation of treatment compared to levels observed at baseline pre-treatment initiation (**Figures 3E, F**). BII-3 is about to start treatment with baricitinib in view of recent development of similar skin lesions. Baricitinib treatment is now also being considered for the affected individuals in family A, should their symptoms worsen.

## Discussion

In this report, we present two families with ISG15 deficiency caused by novel deleterious genetic variants. In family A, we identified an ISG15 variant (p.Q16X) causing premature termination, alongside a multigene 1p36.33 region deletion encompassing *ISG15*. Family B’s affected siblings had homozygous deletion of the entire *ISG15* gene with biparental inheritance. We confirmed enhanced IFN-α/β immunity in all affected cases, as evidenced by upregulated ISG expression, elevated circulating interferons and other cytokines, and activation of p-STAT1 in lymphocytes. Notably, we report for the first time the successful use of JAK inhibition therapy to treat ISG15 deficiency.

ISG15 deficiency is exceedingly rare. Initially linked to mycobacterial susceptibility (4), subsequent reports highlighted diverse cellular, immunological, and clinical manifestations like ulcerative skin lesions, cerebral calcification, and lung inflammation consistent with enhanced IFN-α/β immunity and resembling other Mendelian autoinflammatory interferonopathies (5, 6, 15, 16). The variability in the clinical presentation of ISG15 deficiency is not unexpected, considering that ISG15 serves as both a redundant factor in antiviral immunity and a negative regulator of IFN-α/β immunity. We observed a remarkable skin phenotype in our patients characterised by severe necrotising skin ulceration. Notably, affected individuals in family B exhibited mild developmental delay and seizures, without evidence of cerebral inflammation or calcification; ongoing neuroimaging surveillance is underway. Additionally, close clinical monitoring has been initiated for AII-2, who is currently asymptomatic.

We emphasise the limitations of short-read sequencing for detecting substantial deletions/duplications and advocate integrating copy number variant (CNV) callers into genetic testing pipelines. Utilising ExomeDepth (12) we were able to detect a 10-gene deletion including *ISG15* in AII-1, confirmed by targeted genomic microarray analysis. In family B, the *ISG15* gene deletion was too large to be called by standard variant calling, but in addition it was too small to be visible through microarray analysis. SavvyCNV (14) and ExomeDepth (12) detected homozygous gene deletion in BII-2 and BII-3, one copy from each parent. We would suggest that for individuals with a highly suspicious clinical presentation for ISG15 deficiency in whom only one or no pathogenic variant is found, performing gene-targeted CNV analysis. There is also a need to consider systematically testing for gene deletions/duplications across all autoinflammatory diseases (17–20).

The pathophysiology underlying the skin lesions observed in ISG15-deficient patients is likely to be complex. We observed elevated levels of p-STAT1 in keratinocytes of the epidermis in a lesional skin biopsy from a patient with ISG15 deficiency. Enhanced IFN-I signaling has been reported in dermal endothelial cells, monocytes, and macrophages (6). Recent evidence also suggests that ISG15 plays a crucial role in maintaining cell migration and epidermal homeostasis (15, 21), which may contribute to the significant scar formation observed in ISG15 deficiency. We also observed significant scarring in all symptomatic ISG15-deficient patients, although we did not investigate the specific mechanisms underlying this process. It would also be of interest to assess the expression of ISG proteins (ISG15 or USP18) in the skin of healthy vs. ISG15-deficient subjects, but this was not possible due to limited availability of skin biopsy material in these cases.

We present, for the first time, data demonstrating the efficacy of targeted therapy with JAK inhibition for the treatment of ISG15 deficiency. Earlier *in vitro* experiments by Malik et al. showed that JAK inhibition effectively reduces hyperinflammation and enhances cell migration in ISG15 deficiency (15). Considering this alongside the favourable outcomes of JAK inhibitors in various interferonopathies (22–29), we treated one ISG15-deficient patient from family B with baricitinib. Baricitinib was well tolerated and led to rapid and sustained clinical improvement. Baricitinib treatment reduced p-STAT1 expression in lymphocytes derived from BII-2 and decreased cytokine levels compared to pre-treatment levels. Our findings suggest that JAK inhibition is a logical and effective therapeutic approach for ISG15 deficiency. We note that emerging data indicate that JAK inhibition may be particularly effective in addressing the systemic and cutaneous manifestations of these interferonopathies, while efficacy in treating neuroinflammation or pulmonary inflammation may be limited (30, 31). Future studies are needed to establish if this is also the case in ISG15 deficiency.

We did not investigate whether the specific *ISG15* variants increased susceptibility to mycobacterial disease. Although BII-2 received empirical treatment for TBM, we did not confirm any mycobacterial infection. It would be interesting to further examine the effects of these ISG15 variants on leukocyte mycobacterium-induced ISG15 secretion and lymphocyte/natural killer cell production of IFN-γ. Another limitation of this study was paucity of blood sample volume, due to both children being difficult to bleed. We therefore acknowledge that some further functional studies may have been of interest but were not possible here.

In conclusion, our study presents two families with ISG15 deficiency, expanding the genetic spectrum of the disease. Furthermore, we demonstrate for the first time that JAK inhibition with baricitinib can be an effective therapy for ISG15 deficiency.

## Data availability statement

The data presented in the study are deposited in the NCBI Sequence Read Archive (SRA), accession number PRJNA1021542, https://www.ncbi.nlm.nih.gov/sra/PRJNA1021542.

## Ethics statement

The studies involving humans were approved by Bloomsbury ethics committee, NHS HRA. The studies were conducted in accordance with the local legislation and institutional requirements. Written informed consent for participation in this study was provided by the participants’ legal guardians/next of kin. Written informed consent was obtained from the individual(s), and minor(s)’ legal guardian/next of kin, for the publication of any potentially identifiable images or data included in this article.

## Author contributions

AB: Conceptualization, Data curation, Investigation, Methodology, Writing – original draft, Writing – review & editing, Visualization. EM: Conceptualization, Investigation, Writing – original draft, Writing – review & editing. EA: Investigation, Writing – original draft, Writing – review & editing. EA-A: Conceptualization, Investigation, Writing – original draft, Writing – review & editing. YH: Conceptualization, Investigation, Methodology, Supervision, Writing – review & editing. EO: Conceptualization, Investigation, Methodology, Supervision, Writing – review & editing. HT: Investigation, Writing – review & editing. KS: Investigation, Writing – review & editing. WJ: Writing – review & editing, Investigation. AG: Investigation, Writing – review & editing. VJ: Investigation, Writing – review & editing. W-LD: Investigation, Methodology, Writing – review & editing. NS: Investigation, Writing – original draft, Writing – review & editing. LS: Investigation, Writing – review & editing. MO: Investigation, Writing – review & editing. SW: Investigation, Writing – review & editing. AS: Investigation, Writing – review & editing. IW: Investigation, Writing – review & editing. FP-K: Data curation, Writing – original draft, Writing – review & editing. BJ: Investigation, Writing – review & editing. PB: Conceptualization, Funding acquisition, Supervision, Writing – original draft, Writing – review & editing. DE: Conceptualization, Data curation, Funding acquisition, Methodology, Supervision, Writing – original draft, Writing – review & editing.

## References

[1] Eleftheriou D Brogan PA. Genetic interferonopathies: An overview. Best Pract Res Clin Rheumatol (2017) 31(4):441–59. doi: 10.1016/j.berh.2017.12.002 29773266

[2] Crow YJ Stetson DB. The type I interferonopathies: 10 years on. Nat Rev Immunol (2021) 22(8):471–83. doi: 10.1038/s41577-021-00633-9 34671122 PMC8527296

[3] Kerner G Rosain J Guérin A Al-Khabaz A Oleaga-Quintas C Rapaport F. Inherited human IFN-γ deficiency underlies mycobacterial disease. J Clin Invest (2020) 130(6):3158–71. doi: 10.1172/JCI135460 PMC7260033 32163377

[4] Bogunovic D Byun M Durfee LA Abhyankar A Sanal O Mansouri D. Mycobacterial disease and impaired IFN-γ Immunity in humans with inherited ISG15 deficiency. Science (2012) 337(6102):1684–8. doi: 10.1126/science.1224026 PMC3507439 22859821

[5] Zhang X Bogunovic D Payelle-Brogard B Francois-Newton V Speer SD Yuan C. Human intracellular ISG15 prevents interferon-α/β over-amplification and auto-inflammation. Nature (2015) 517(7532):89–93. doi: 10.1038/nature13801 25307056 PMC4303590

[6] Martin-Fernandez M Bravo García-Morato M Gruber C Murias Loza S Malik MNH Alsohime F. Systemic type I IFN inflammation in human ISG15 deficiency leads to necrotizing skin lesions. Cell Rep (2020) 31(6):107633. doi: 10.1016/j.celrep.2020.107633 32402279 PMC7331931

[7] Li H Durbin R. Fast and accurate short read alignment with Burrows-Wheeler transform. Bioinformatics (2009) 25(14):1754–60. doi: 10.1093/bioinformatics/btp324 PMC2705234 19451168

[8] Yang H Wang K. Genomic variant annotation and prioritization with ANNOVAR and wANNOVAR. Nat Protoc (2015) 10(10):1556–66. doi: 10.1038/nprot.2015.105 PMC4718734 26379229

[9] Richards S Aziz N Bale S Bick D Das S Gastier-Foster J. Standards and guidelines for the interpretation of sequence variants: a joint consensus recommendation of the American College of Medical Genetics and Genomics and the Association for Molecular Pathology. Genet Med (2015) 17(5):405–23. doi: 10.1038/gim.2015.30 PMC4544753 25741868

[10] Ellard S Baple EL Callaway A Berry I Forrester N Turnbull C. ACGS best practice guidelines for variant classification in rare disease (2020). ACGS website. Available at: ttps://www.acgs.uk.com/media/11631/uk-practice-guidelines-for-variant-classification-v4-01-2020.pdf.

[11] Smedley D Jacobsen JOB Jäger M Köhler S Holtgrewe M Schubach M. Next-generation diagnostics and disease-gene discovery with the Exomiser. Nat Protoc (2015) 10(12):2004–15. doi: 10.1038/nprot.2015.124 PMC5467691 26562621

[12] Plagnol V Curtis J Epstein M Mok KY Stebbings E Grigoriadou S. A robust model for read count data in exome sequencing experiments and implications for copy number variant calling. Bioinformatics (2012) 28(21):2747–54. doi: 10.1093/bioinformatics/bts526 PMC3476336 22942019

[13] Chen W Rehsi P Thompson K Yeo M Stals K He L. Clinical and molecular characterization of novel FARS2 variants causing neonatal mitochondrial disease. Mol Genet Metab (2023) 140(3):107657. doi: 10.1016/j.ymgme.2023.107657 37523899

[14] Laver TW De Franco E Johnson MB Patel KA Ellard S Weedon MN. SavvyCNV: Genome-wide CNV calling from off-target reads. PloS Comput Biol (2022) 18(3):e1009940. doi: 10.1371/journal.pcbi.1009940 35294448 PMC8959187

[15] Malik MNH Waqas SF-U-H Zeitvogel J Cheng J Geffers R Gouda ZA-E. Congenital deficiency reveals critical role of ISG15 in skin homeostasis. J Clin Invest (2022) 132(3):e141573. doi: 10.1172/JCI141573 34847081 PMC8803340

[16] Buda G Valdez RM Biagioli G Olivieri FA Affranchino N Bouso C. Inflammatory cutaneous lesions and pulmonary manifestations in a new patient with autosomal recessive ISG15 deficiency case report. Allergy Asthma Clin Immunol (2020) 16(1):77. doi: 10.1186/s13223-020-00473-7 32944031 PMC7491304

[17] Li GM Han X Wu Y Wang W Tang HX Lu MP. A cohort study on deficiency of ADA2 from China. J Clin Immunol (2023) 43(4):835–45. doi: 10.1007/s10875-023-01432-8 PMC10110724 36807221

[18] Zhang C Han X Sun L Yang S Peng J Chen Y. Novel loss-of-function mutations in TNFAIP3 gene in patients with lupus nephritis. Clin Kidney J (2022) 15(11):2027–38. doi: 10.1093/ckj/sfac130 PMC9613433 36325013

[19] Ripen AM Chiow MY Rama Rao PR Mohamad SB. Revealing chronic granulomatous disease in a patient with williams-beuren syndrome using whole exome sequencing. Front Immunol (2021) 12:778133. doi: 10.3389/fimmu.2021.778133 34804071 PMC8599285

[20] Reddy S Jia S Geoffrey R Lorier R Suchi M Broeckel U. An autoinflammatory disease due to homozygous deletion of the IL1RN locus. N Engl J Med (2009) 360(23):2438–44. doi: 10.1056/NEJMoa0809568 PMC2803085 19494219

[21] Waqas SFUH Sohail A Nguyen AHH Usman A Ludwig T Wegner A. ISG15 deficiency features a complex cellular phenotype that responds to treatment with itaconate and derivatives. Clin Trans Med (2022) 12(7):e931. doi: 10.1002/ctm2.931 PMC9288839 35842904

[22] Crow YJ Neven B Frémond M-L. JAK inhibition in the type I interferonopathies. J Allergy Clin Immunol (2021) 148(4):991–3. doi: 10.1016/j.jaci.2021.07.028 34375617

[23] Frémond ML Rodero MP Jeremiah N Belot A Jeziorski E Duffy D. Efficacy of the Janus kinase 1/2 inhibitor ruxolitinib in the treatment of vasculopathy associated with TMEM173-activating mutations in 3 children. J Allergy Clin Immunol (2016) 138(6):1752–5. doi: 10.1016/j.jaci.2016.07.015 27554814

[24] Higgins E Al Shehri T McAleer MA Conlon N Feighery C Lilic D. Use of ruxolitinib to successfully treat chronic mucocutaneous candidiasis caused by gain-of-function signal transducer and activator of transcription 1 (STAT1) mutation. J Allergy Clin Immunol (2015) 135(2):551–3. doi: 10.1016/j.jaci.2014.12.1867 25662309

[25] König N Fiehn C Wolf C Schuster M Cura Costa E Tüngler V. Familial chilblain lupus due to a gain-of-function mutation in STING. Ann Rheum Dis (2017) 76(2):468–72. doi: 10.1136/annrheumdis-2016-209841 27566796

[26] Seo J Kang JA Suh DI Park EB Lee CR Choi SA. Tofacitinib relieves symptoms of stimulator of interferon genes (STING)-associated vasculopathy with onset in infancy caused by 2 *de novo* variants in TMEM173. J Allergy Clin Immunol (2017) 139(4):1396–9.e12. doi: 10.1016/j.jaci.2016.10.030 28041677

[27] Vargas-Hernández A Mace EM Zimmerman O Zerbe CS Freeman AF Rosenzweig S. Ruxolitinib partially reverses functional natural killer cell deficiency in patients with signal transducer and activator of transcription 1 (STAT1) gain-of-function mutations. J Allergy Clin Immunol (2018) 141(6):2142–55.e5. doi: 10.1016/j.jaci.2017.08.040 29111217 PMC5924437

[28] Weinacht KG Charbonnier LM Alroqi F Plant A Qiao Q Wu H. Ruxolitinib reverses dysregulated T helper cell responses and controls autoimmunity caused by a novel signal transducer and activator of transcription 1 (STAT1) gain-of-function mutation. J Allergy Clin Immunol (2017) 139(5):1629–40.e2. doi: 10.1016/j.jaci.2016.11.022 28139313 PMC5482293

[29] Alsohime F Martin-Fernandez M Temsah M-H Alabdulhafid M Le Voyer T Alghamdi M. JAK inhibitor therapy in a child with inherited USP18 deficiency. New Engl J Med (2020) 382(3):256–65. doi: 10.1056/NEJMoa1905633 PMC7155173 31940699

[30] Frémond ML Hully M Fournier B Barrois R Lévy R Aubart M. JAK inhibition in aicardi-goutières syndrome: a monocentric multidisciplinary real-world approach study. J Clin Immunol (2023) 43(6):1436–47. doi: 10.1007/s10875-023-01500-z 37171742 PMC10175907

[31] Frémond ML Hadchouel A Berteloot L Melki I Bresson V Barnabei L. Overview of STING-associated vasculopathy with onset in infancy (SAVI) among 21 patients. J Allergy Clin Immunol Pract (2021) 9(2):803–18.e11. doi: 10.1016/j.jaip.2020.11.007 33217613

